# JAG1 Is Associated with Poor Survival through Inducing Metastasis in Lung Cancer

**DOI:** 10.1371/journal.pone.0150355

**Published:** 2016-03-01

**Authors:** Wen-Hsin Chang, Bing-Ching Ho, Yi-Jing Hsiao, Jin-Shing Chen, Chien-Hung Yeh, Hsuan-Yu Chen, Gee-Chen Chang, Kang-Yi Su, Sung-Liang Yu

**Affiliations:** 1 Institute of Molecular Medicine, College of Medicine, National Taiwan University, Taipei, Taiwan; 2 Department of Clinical Laboratory Sciences and Medical Biotechnology, College of Medicine, National Taiwan University, Taipei, Taiwan; 3 Division of Thoracic Surgery, National Taiwan University Hospital and National Taiwan University College of Medicine, Taipei, Taiwan; 4 Department of Surgery, National Taiwan University Hospital and National Taiwan University College of Medicine, Taipei, Taiwan; 5 Institute of Statistical Science, Academia Sinica, Taipei, Taiwan; 6 Faculty of Medicine, School of Medicine, National Yang-Ming University, Taipei, Taiwan; 7 Comprehensive Cancer Center, Taichung Veterans General Hospital, Taichung, Taiwan; 8 Department of Laboratory Medicine, National Taiwan University Hospital, Taipei, Taiwan; 9 Department of Pathology, College of Medicine, National Taiwan University, Taipei, Taiwan; 10 Graduate Institute of Pathology, College of Medicine, National Taiwan University, Taipei, Taiwan; 11 Center for Optoelectronic Biomedicine, College of Medicine, National Taiwan University, Taipei, Taiwan; 12 Institute of Medical Device and Imaging, College of Medicine, National Taiwan University, Taipei, Taiwan; H. Lee Moffitt Cancer Center & Research Institute, UNITED STATES

## Abstract

JAG1 is a Notch ligand that plays a critical role in multiple signaling pathways. However, the functionality of JAG1 in non-small cell lung cancer (NSCLC) has not been investigated thoroughly. By comparison of gene transcripted RNA profiles in the cell line pair with differential invasion ability, we identified JAG1 as a potential metastasis enhancer in lung cancer. Ectopic expression of JAG1 on lung cancer cells enhanced cell migration and invasion as well as metastasis *in vitro* and *in vivo*. Conversely, knockdown of JAG1 with siRNA in highly invasive cancer cells led to the reduction of migration and invasion. In clinical analysis, JAG1 mRNA expression was higher in tumors than in adjacent normal tissues in 14 of 20 patients with squamous cell carcinoma (SCC). SCC patients with higher JAG1 transcription had poor overall survival than those with low-transcripted JAG1. Microarray analysis indicated that the enforced JAG1 transcription was associated with an elevated HSPA2 RNA transcription, which played a role in promoting cancer cell migration and invasion. In conclusion, this is the first study that demonstrated that JAG1 might act as a potential prognostic marker and JAG1/HSPA2 axis mediates lung cancer malignancy at least partly.

## Introduction

Lung cancer is the leading cause of cancer-related death worldwide [1]. Metastasis is a major determinant contributing to cancer progression and mortality, and cell motility and invasion is the initial step [2]. Generally, most cancer can be attributed to a growing number of genetic alterations, which include oncogene activation or tumor suppressor gene inactivation [3, 4]. In our previous studies, we established a series of lung cancer cell lines with varied invasiveness [5] and we identified and validated several oncogenes or tumor suppressors from these model cell lines [6–8]. The discovery of these alterations may benefit cancer patients as cancer markers or druggable targets [9]. However, most alternations are yet to be elucidated completely.

JAG1 is one of the canonical ligands for Notch receptors. After the interaction of ligands with receptors, Notch receptors undergo proteolytic cleavages, and the Notch intracellular domain translocate into the nucleus and turns on the expression of Notch target genes, resulting in cell differentiation, proliferation, survival and apoptosis [10]. In human disease, JAG1 has been reported to be associated with Alagille syndrome [11, 12]. Furthermore, JAG1 is highly expressed in medulloblastoma and colorectal cancer [13, 14], and causes poorer overall survival in breast cancer [15]. In non-small cell lung cancer, JAG1 has been identified in a nine gene-signatures which can possibly be used as genetic markers [16]. In spite of these clinical observations, the molecular contribution and functionality of JAG1 in neoplastic diseases remain to be established.

In this study, we explored the impact of JAG1 on lung cancer invasion both *in vitro* and *in vivo*. The microarray analysis was used to investigate the possible downstream signaling of JAG1. Clinically, the correlation of JAG1 transcription and the survivals of NSCLC were also analyzed.

## Materials and Methods

### Cell culture

CL1-0 and CL1-5 cells were established and characterized as previously described [5]. A549 and H226 were purchased from the American Type Culture Collection (ATCC). Cells were cultured in RPMI-1640 medium with 10% fetal bovine serum and 1% penicillin-streptomycin (Invitrogen, Carlsbad, CA) at 37°C in a humidified atmosphere of 5% CO_2_.

### Plasmid construction, transfection and stable clones

The JAG1 and HSPA2 expressing plasmids, pcDNA3.1-JAG1-V5 and pcDNA3.1-HSPA2-V5, were constructed by cloning the full-length human JAG1 or HSPA2 cDNA with C-terminal V5 tag into the pcDNA3.1 vector (Life Technologies, Grand Island, NY), respectively. Two siRNA used for knockdown JAG1 mRNA were purchased from Silencer^®^ Select siRNA (Cat. s1175 and s1176) (Life Technologies, Grand Island, NY). All transfection experiments were performed with Lipofectamine^®^ 2000 reagent (Invitrogen, Carlsbad, CA) in accordance with the manufacturer’s protocol. CL1-0 cells were transfected with pcDNA3.1-JAG1 or pcDNA3.1 empty vector. After culturing in medium containing 1 mg/mL of geneticin (G418) for 2–3 weeks, pooled stable clones were isolated. Clones that express the JAG1 cDNA coding region were maintained in medium containing 0.5 mg/mL of geneticin and used for further investigation.

### Real-time quantitative RT-PCR

Total RNA was isolated by the TRIzol reagent (Life Technologies, Grand Island, NY), and 1 μg of total RNA was used in cDNA synthesis with random hexamer primers using Superscript II reverse transcriptase (Life Technologies, Grand Island, NY). 20 ng RNAs or 10 ng cDNAs were served as the template for the mRNA expression detection by real-time quantitative PCR with SYBER Green. The relative mRNA expression level of target gene was determined as -ΔCT = -[CT_target_-CT_TBP_]. The target/TBP mRNA ratio was calculated as 2-ΔCT × K, in which K is a constant. The detail primer sequence for each target gene real-time quantitative PCR was listed in S1 Table.

### Immunoblotting

The preparation of whole-cell lysates and Western blot analysis were described as previously mentioned [7, 8]. Briefly, the cell lysates for immunoblot were prepared in RIPA buffer (1% NP-40, 0.5% sodium deoxycholate, 0.1% SDS, 50mM Tris-HCl, pH 7.5) containing 1X complete protease inhibitor cocktail (Roche, Indianapolis, IN). The protein samples were separated by 8%~12% SDS-PAGE, and transferred to a poly (vinylidene difluoride) membrane (Merck Millipore, Billerica, MA). Proteins were probed with specific antibodies, visualized by chemiluminescence assay kit (Merck Millipore, Billerica, MA) and detected by FUJIFILM LAS-3000 ECL system (GE Healthcare, Piscataway, NJ). Primary antibodies used for immunoblot were as follows: anti-JAG1, anti-NICD-3 (Santa Cruz Biotechnology, Santa Cruz, CA), anti-NICD-1, anti-NICD-2, anti-NICD-4 (GeneTex, Irvine, CA), anti-β-actin (Sigma, St Louis, MO), and anti-V5 (Invitrogen, Carlsbad, CA). The fluorescent immunohistochemistry staining in patients’ tumor biopsy was descripted in S1 Text.

### Migration and invasion assays

*In vitro* cell migration and invasion assays were performed as previously described [17] using transwell chambers (8 μm pore size; Costar, Cambridge, MA). In migration assay, 1 × 10^5^ cells were seeded on top of the polycarbonate filters and incubated for 8 hours. Filters were swabbed with a cotton swab, fixed with methanol and then stained with Giemza solution (Sigma, St Louis, MO). For the invasion assay, filters were coated with Matrigel (Becton Dickinson, Franklin Lakes, NJ), and 1 × 10^5^ cells were seeded onto the Matrigel and incubated for 18 hours. The cells attached to the lower surface of the filter were counted under a light microscope (Magnification ×100).

### *In vivo* metastasis

The animal study was approved by the Institutional Animal Care and Use Committee of National Taiwan University with IACUC No. 20080239. For metastasis assay [7], 1×10^6^ cells were washed and resuspended in 100 μl PBS. Subsequently, cells were injected into the lateral tail vein of 6-week-old SCID mice (supplied by the Laboratory Animal Center of the College of Medicine, National Taiwan University, Taipei, Taiwan). After implantation of 10 weeks, the lungs of the mice were removed and fixed in 10% formalin and the number of lung micrometastatic lesions was counted under a dissecting microscope. Embedded tissues were sliced and stained with hematoxylin-eosin for histological analysis.

### Patients and tissue specimens

This investigation was performed after approval by the Institutional Review Board of National Taiwan University Hospital with NTUH-REC No. 200812077R protocol. Written informed consent was obtained from all patients. All of the tumor tissue samples were treatment naive because none of the patients had received neo-adjuvant chemotherapy or radiation therapy before surgery. Specimens of lung cancer tissue and the non-tumor part of the lung obtained at surgery were immediately snap-frozen in liquid nitrogen and stored at -80°C until use. The histologic classification of these tumors was based on the World Health Organization criteria. Tumor size, local invasion, and lymph node metastasis were determined at pathologic examination. The final staging of the disease was determined from a combination of surgical and pathologic findings, according to the current tumor-node-metastasis system for lung cancer staging. Follow-up data will be obtained from the patients’ medical charts and reported from our tumor registry service. Relapse time was calculated from the date of operation to the date of detection of local recurrence or systemic metastasis. Survival time was calculated from the date of operation to the date of death. Patients dying of post-operative complications within 30 days after surgery were excluded from survival analysis, so as to avoid bias. The analysis of JAG1 mRNA expression in patients was descripted in S1 Text.

### Microarray analysis

For cDNA microarray analysis, cDNA preparation and array hybridization were performed according to the Affymetrix GeneChip Expression Analysis Technical Manual. Briefly, the 8 μg total RNA was reverse-transcribed in the presence of a T7 primer (One-cycle cDNA Synthesis kit; Affymetrix, Santa Clara, CA). The cDNA product was purified and transcribed *in vitro* with biotin-labeled ribonucleotides (IVT Labeling Kit; Affymetrix, Santa Clara, CA). A portion of the biotinylated RNA was fragmented and hybridized overnight to Human genome U133 plus 2.0 GeneChip (Affymetrix, Santa Clara, CA). The GeneChip was washed and developed by the amplification staining protocol provided by Affymetrix. The GeneChip was scanned by Affymetrix GeneChip Scanner 3000 7G, and the images were extracted with Affymetrix GeneChip Operating Software (GCOS) version 1.4. All hybridization experiments were performed in biological triplicate with cDNA probes prepared from CL1-0/JAG1 and CL1-0/vector control. Data were filtered by 2-fold changes under FDR protection (*p*<0.05) using Genespring GX 12.6 (Sillicon Genetics, Redwood City, CA). The microarray CEL files, normalized data and experimental information have been deposited in the Gene Expression Omnibus, and are available by the accession number GSE14995. The detail experimental procedure and followed identification of JAG1 downstream genes was descripted in S1 Text.

### Statistical analysis

All experiments are performed in triplicate and analyzed by ANOVA (Excel, Microsoft; Taipei, Taiwan). *p* value < 0.05 is considered statistically significant. Data are presented as the mean ± SD.

## Results

### JAG1 is identified as a potential metastasis-promoting gene

**Fig 1:**
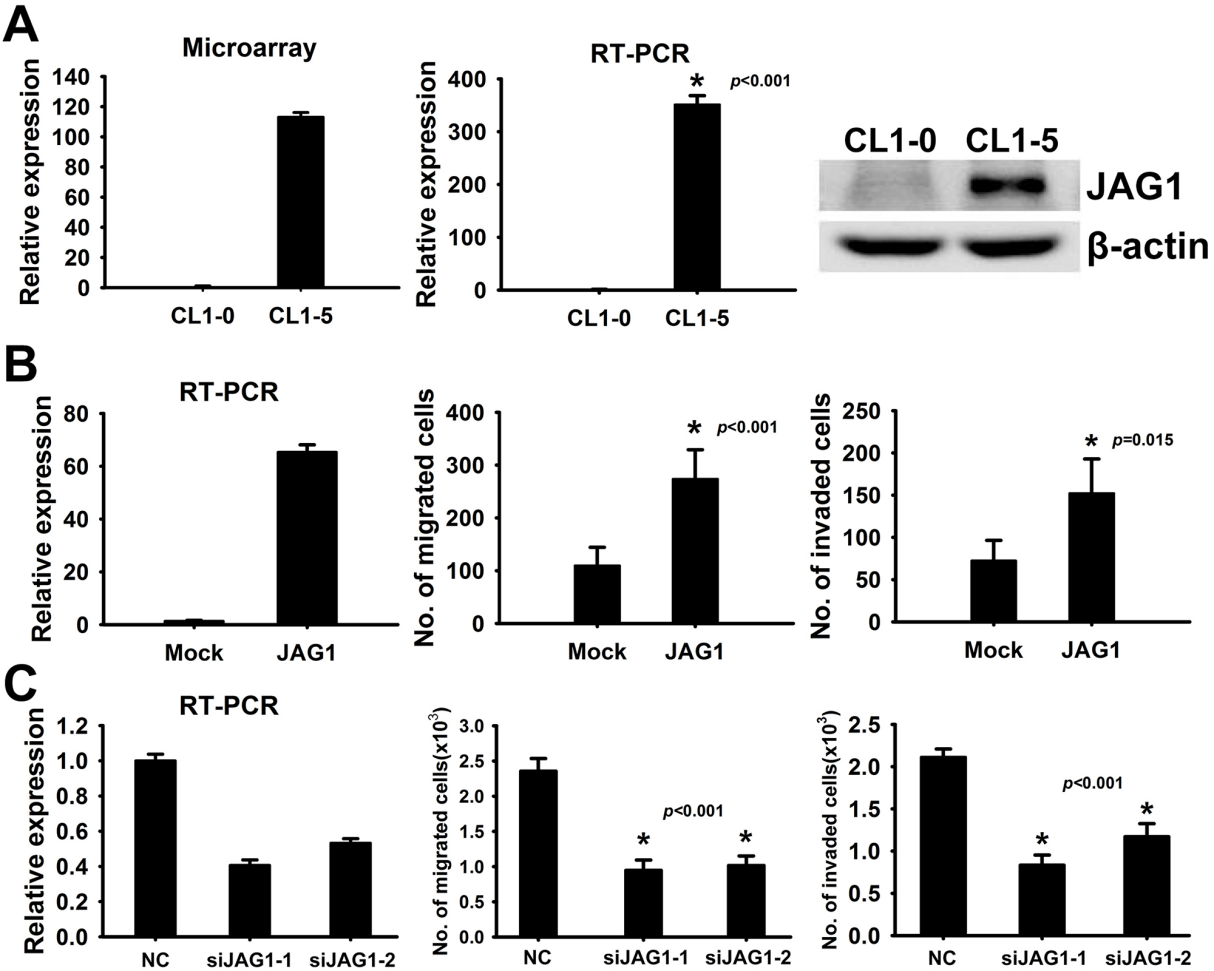
JAG1 acts as an invasion-promoting gene. (A) Identification of JAG1 in an invasion model of lung cancer cells. JAG1 is up-regulated in highly invasive lung cancer cells. Left, JAG1 mRNA level was assessed by oligonucleotide microarray analysis. Middle, JAG1 mRNA level was measured by real-time quantitative RT-PCR. The experiments were performed in triplicate and data were represented as mean ± SD. Right, JAG1 protein expression was evaluated by Western blot. β-actin was used as an internal control. (B) JAG1 promotes cell migration and invasiveness *in vitro*. Left, JAG1 was stably overexpressed in CL1-0 cells. JAG1 mRNA was measured by real-time quantitative RT-PCR in triplicate. Data were represented as mean ± SD. Middle and Right, migration and invasion ability of CL1-0 cells with constitutive JAG1 expression and mock control cells were evaluated by migration assay and matrigel invasion assay. Data presented as mean ± SD of three experiments. *: *p* < 0.05 as compared to the mock group. (C) Knockdown of JAG1 inhibits cell migration and invasiveness *in vitro*. Left, JAG1 mRNA was knocked down by siRNAs in CL1-5 cells assayed by real-time quantitative RT-PCR in triplicate. Two different siRNAs (siJAG1-1 and siJAG1-2) were used to silence JAG1. NC, negative control. Middle and Right, migration and invasion capability of JAG1-silencing transfectants were analyzed by migration and matrigel invasion assays. Data shown as mean ± SD; *: *p* < 0.05 compared with the negative control.

To identify the novel genes involved in lung cancer metastasis, cDNA expression microarrays were used to study a lung cancer invasion model in our previous studies [18]. After comparison of mRNA expression profiles of the low invasive cells (CL1-0) and the highly invasive cells (CL1-5), we found the mRNA expression of JAG1 was 113-fold higher in CL1-5 than in CL1-0 (Fig 1A, left). The transcript expression and protein expression of JAG1 in both the CL1-0 and CL1-5 cell lines were consistent with the results obtained from microarray analysis (Fig 1A, middle and right). To verify whether JAG1 plays a critical role in regulating cancer metastasis, we used overexpression and RNAi silencing approaches to modulate JAG1 transcriptional expression and performed transwell migration and invasion assays. As shown in Fig 1B and 1C, enforced expression of JAG1 promoted the ability of cell migration and invasion in the low invasive CL1-0 cells. In contrast, knockdown of JAG1 inhibited both activities in highly invasive CL1-5 cells. These data suggested that JAG1 might act as a novel candidate involved in acceleration of lung cancer metastasis.

### JAG1 promotes NSCLC malignancy

**Fig 2:**
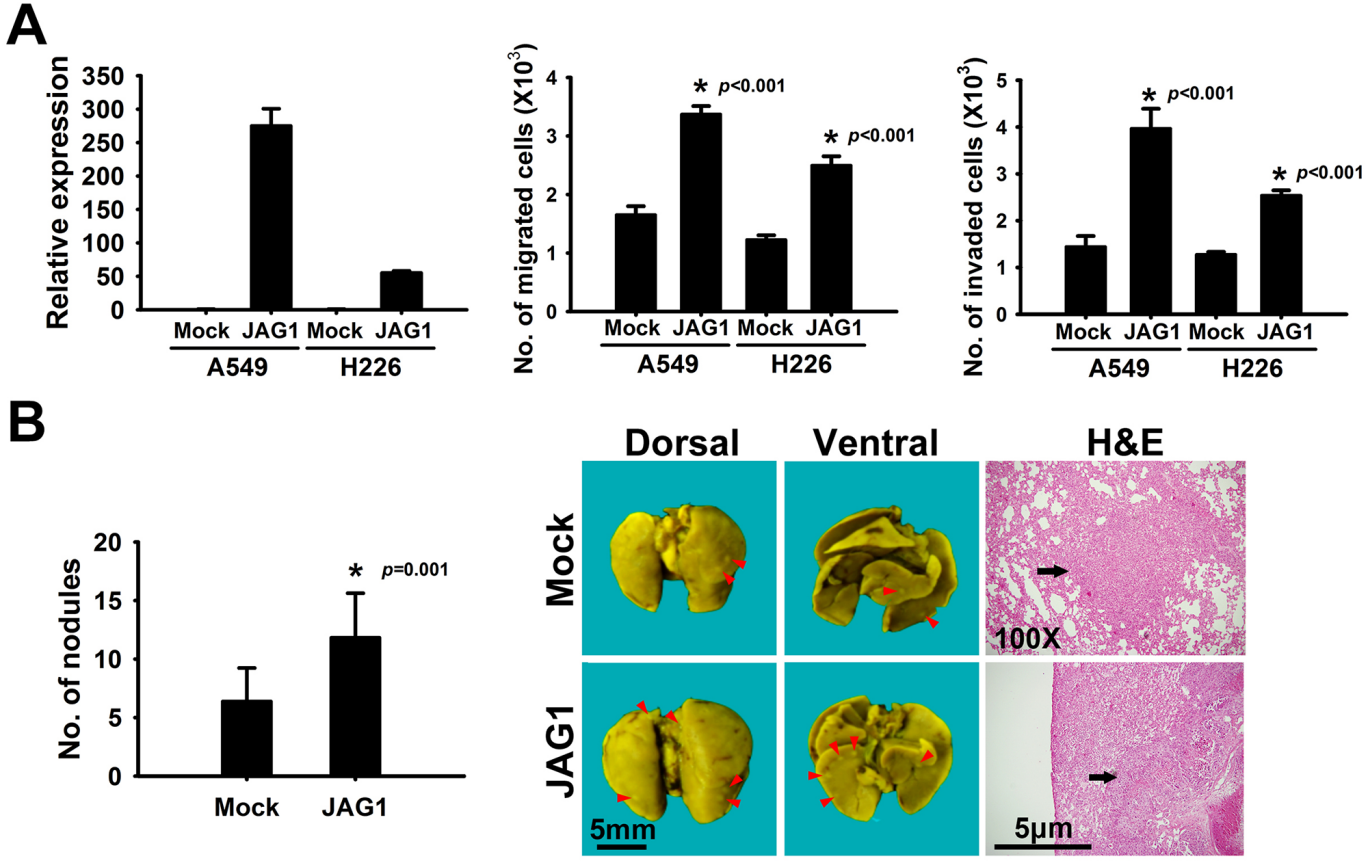
JAG1 enhances migration and invasion in different lung cancer cell lines and promotes metastasis *in vivo*. (A) JAG1 promotes cell migration and invasiveness *in vitro*. Left, JAG1 was transiently overexpressed in A549 and H226 cells. JAG1 mRNA was measured by real-time quantitative RT-PCR and normalized to TBP in triplicate. Middle and Right, migration ability and invasiveness of cells with transient JAG1 overexpression and control cells were evaluated by migration and matrigel invasion assays. *: *p* < 0.05, compared with mock. Data were represented as mean ± SD in triplicate. (B) JAG1 promotes metastasis *in vivo*. Left, number of nodules in SCID mice. The Mock CL1-0 cells and JAG1-overexpressing cell lines were inoculated into severe combined immunodeficiency (SCID) mice by tail vein injection. After 10 weeks, mice were sacrificed. Number of tumors derived from mock and JAG1 transfectants was measured under the dissection microscope. Data were represented as mean ± SD (n = 10 in mock control group and n = 13 in JAG1 transfectant group). *: *p* < 0.05, compared with mock control. Right, appearances of the lungs from mice injected with CL1-0 mock and JAG1 transfectants and the representative H&E staining sections of the lungs from mice. Red arrow heads indicate metastatic tumor nodules in lungs. Black arrows indicate the micrometastasis of lung cancer cells.

To confirm whether the oncogenic nature of JAG1 is a universal phenomenon, we performed the transwell migration and invasion assays in other two cell lines. This phenomenon was recapitulated in other non-small cell lung cancer cell lines. We found that transiently enforced expression of JAG1 promoted migrating and invasive abilities *in vitro* not only in CL1-0 but also in A549 and H226 cells (Fig 2A). To confirm whether JAG1 functions in promoting metastasis *in vivo*, CL1-0 cells were transfected with JAG1-expressing or empty vector and were intravenously introduced into NOD SCID mice by tail injection for evaluating their metastasis abilities. After ten weeks of injection, all mice were sacrificed and the metastatic tumor foci in lungs were examined and counted. Mice injected with JAG1 transfectants (n = 13) developed more pulmonary metastasis nodules than mock transfectants (n = 10) (Fig 2B, left and middle). Metastatic lesions in randomly selected lung sections were stained by hematoxylin and eosin to histologically confirm tumor nodules (Fig 2B, right). These results further confirmed that JAG1 plays a critical role in modulating cancer metastasis.

### Association of JAG1 mRNA expression and clinical outcomes

**Fig 3:**
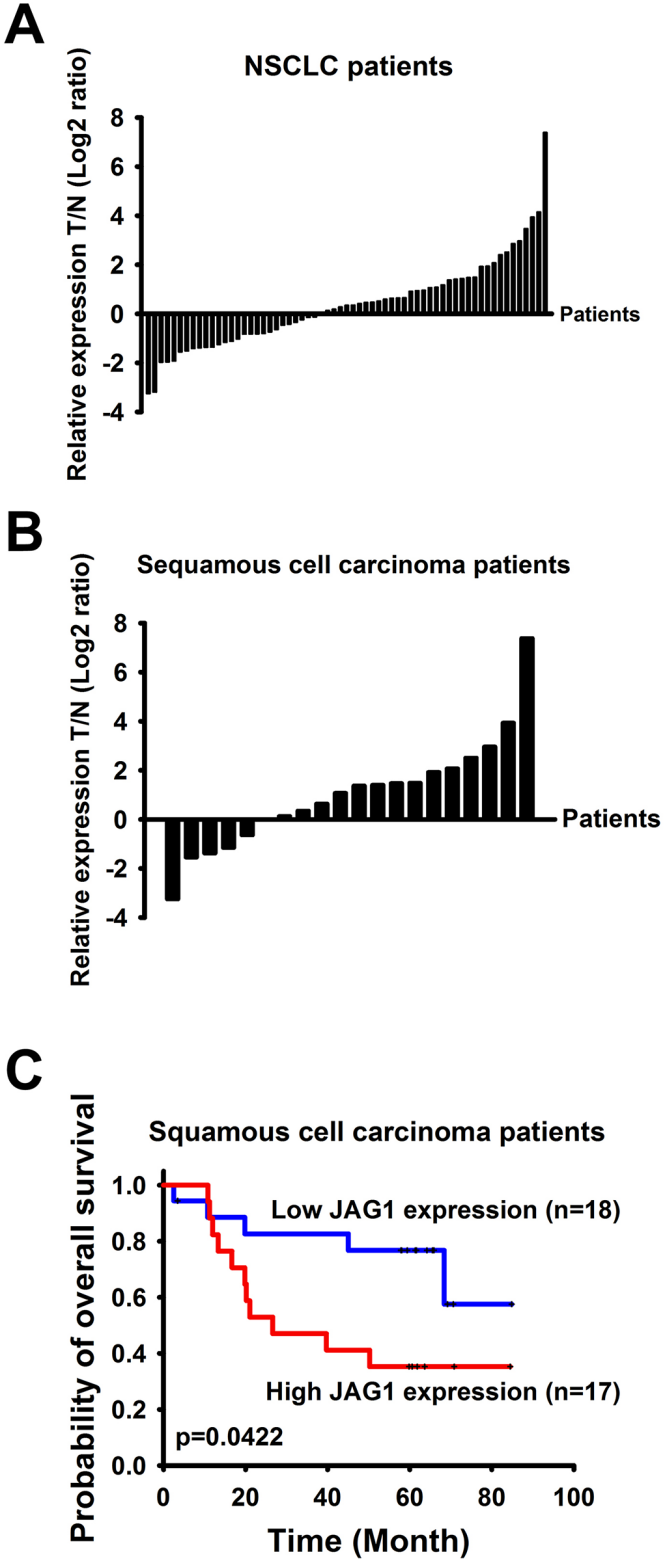
JAG1 expression and NSCLC patients’ survival. (A and B) JAG1 mRNA expression in tumor and normal parts of lung cancer. JAG1 mRNA in tumors and adjacent normal tissues of NSCLC (A) and subtype squamous carcinoma patients (B) was assessed by real-time quantitative RT-PCR and normalized to TBP. (C) JAG1 mRNA level and overall survival of subtype squamous carcinoma patients. All statistical tests were two sided, and *p* <0.05 was considered to be statistically significant.

Given the significance of JAG1 in cancer invasion *in vitro* and *in vivo*, we further examined the impact of JAG1 on the progress of lung cancers. First JAG1 mRNA expression was measured in 63 paired adjacent normal and NSCLC tissues by real-time quantitative RT-PCR. Although the *p*-value did not reach statistical significance (*p* = 0.058), the JAG1 mRNA had a tendency that it was abundant in tumor tissue rather than in adjacent paired normal tissue (Fig 3A). Next, we found that JAG1 was inclined to express in tumor parts compared with adjacent normal tissues of 14 out of 20 squamous carcinoma (*p* = 0.017, Fig 3B). Furthermore, we investigated whether JAG1 mRNA expression is correlated with the survival of patients. Among 90 NSCLC patients, the mRNA level of JAG1 was not correlated to patients’ overall survival (S1 Fig, *p* = 0.9710). However, even though were only 35 patients with squamous cell carcinoma among 90 NSCLC patients, low level mRNA of JAG1 had shown longer overall survival in squamous carcinoma patients than patients with higher level mRNA of JAG1 shown by Kaplan-Meier survival analyses and log-rank tests (*p* = 0.042, Fig 3C). Multivariable Cox proportional hazards regression analysis showed that JAG1 transcription is associated with overall survival of squamous carcinoma (patients with high versus low JAG1 mRNA level, hazard ratio [HR] = 2.87, 95% CI = 0.99 to 8.33; *p* = 0.05).

### HSPA2 is a novel downstream effector of JAG1 in NSCLC

**Fig 4:**
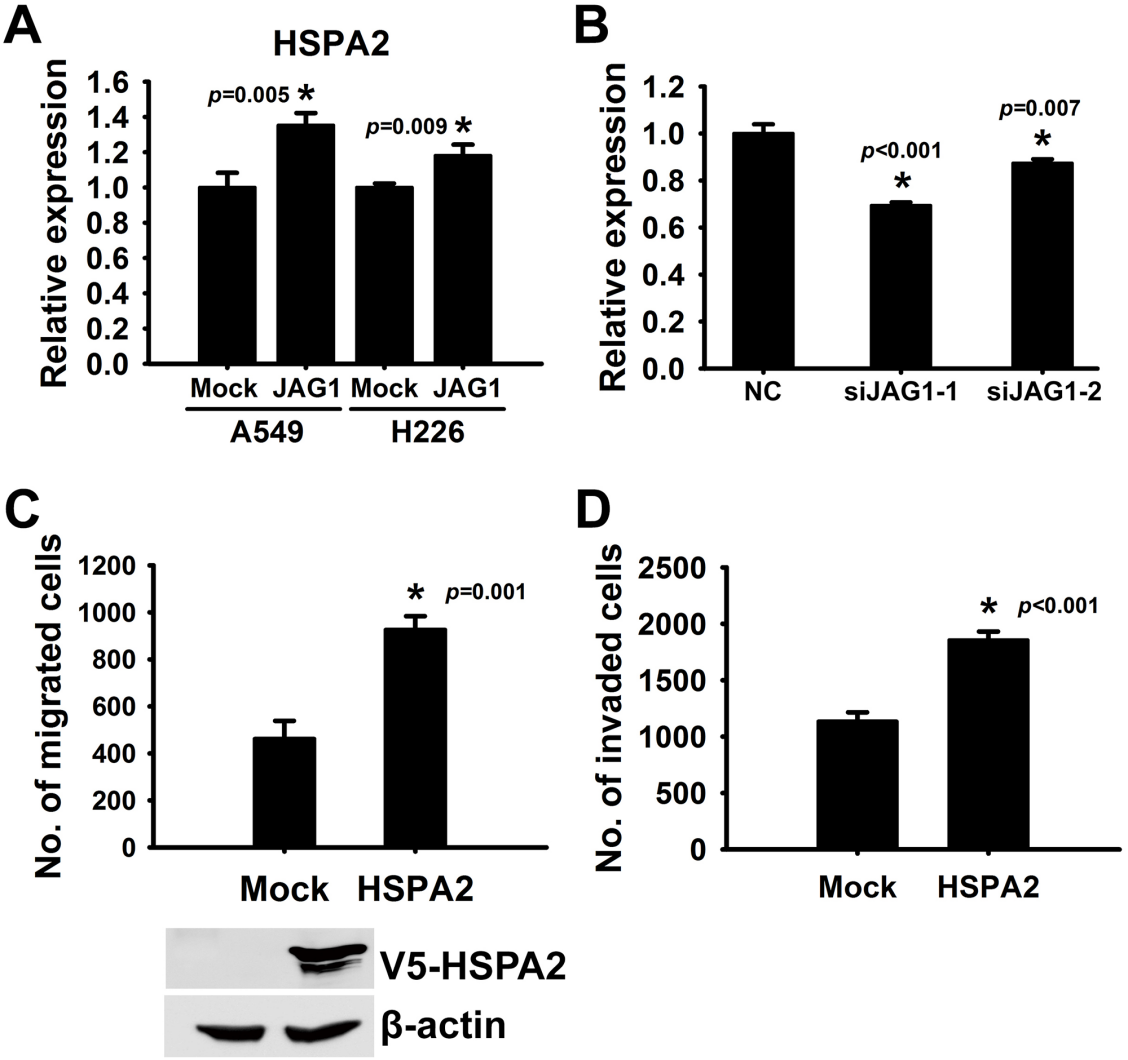
HSPA2 mRNA expression is regulated by JAG1. (A and B) JAG1 was transiently overexpressed in A549 and H226 (A) or knockdown by JAG1 siRNAs in CL1-5 (B). HSPA2 mRNA was measured by real-time quantitative RT-PCR and normalized to TBP. (C) Migration ability of CL1-0 cells with transient HSPA2 overexpression and control cells was evaluated by migration assay. *, *p* <0.05, compared with mock control. Data were represented as mean ± SD (n = 3 per group). (D) Invasiveness of CL1-0 cells with transient HSPA2 overexpression and control cells as evaluated by matrigel invasion assay. *, *p* <0.05, compared with mock control. Data were represented as mean ± SD (n = 3 per group).

**Fig 5:**
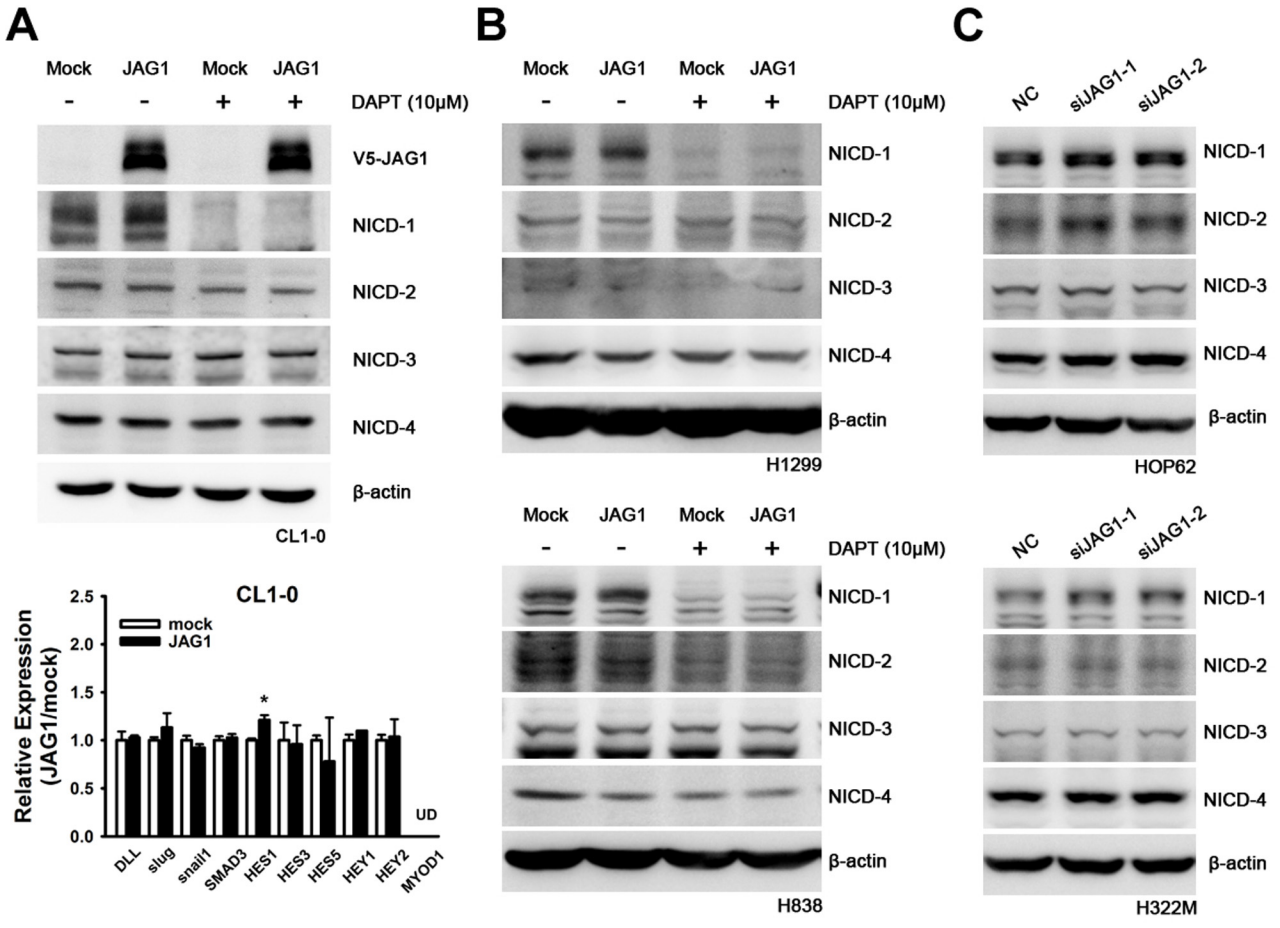
Proteolytic status of NOTCH family or downstream molecules changes in JAG1 manipulated lung cancer cell lines. Cell was transiently overexpressed JAG1 by expression plasmid or silenced JAG1 by siRNA strategy followed by immunoblotted with NICD1, NICD2, NICD3, and NICD4 antibodies or real-time quantitative PCR for NOTCH downstream molecules mRNA level analysis. (A) JAG1 overexpressed in CL1-0 cells followed by Western blot analysis for NICDs proteolytic status or real-time quantitative PCR for NOTCH downstream molecules mRNA level analysis. (B) JAG1 overexpressed in H1299 and H838 cell lines followed by Western blot analysis for NICDs proteolytic status. (C) JAG1 silenced in HOP62 and H322M cell lines followed by Western blot analysis for NICDs proteolytic status. DAPT is a gamma-secretase inhibitor and used for NOTCH signaling control. UD, undetermined due to undetectable expression level; *, *p* <0.05 compared with mock control. Data were represented as mean ± SD (n = 3 per group).

Finally, we utilized the Affymetrix oligonucleotide microarray analysis to identify the top 10 differentially expressed genes in JAG1-tranfected CL1-0 cells compared with the mock transfectant (S2 and S3 Tables). Notably, we did not observe any significant difference of Notch1 and Notch2 and its downstream genes (ASCL1, HES1, HEY1, and SLUG) mRNA level, except trace levels difference of Notch3 and Notch4 RNA level in NSCLC cells (S4 and S5 Tables). In order to identify novel potential JAG1 downstream molecules, we firstly performed RT-PCR to validate genes with significant expression changes analyzed by the microarray result either in JAG1 transfectants or JAG1 silenced CL1-0, CL1-5, A549 and H226 cell lines (S3 Table). Among all genes, HSPA2 exhibited consistent and anticipated tendency among these 10 genes when manipulating JAG1. HSPA2, whose cellular functions in lung cancer are unclear, was higher expressed (1.4 fold in A549 cells and 1.2 fold in H226 cells) in JAG1 transfectants compared with control cells (Fig 4A). Contrariwise, the mRNA expression of HSPA2 was reduced in JAG1 knockdown cells (Fig 4B). In addition, overexpression of V5-HSPA2 in CL1-0 increased cell migration and invasion abilities (Fig 4C and 4D). In order to exclude cell line effect, we further confirmed this phenomenon in more cell lines. Among screened six lung cancer cell lines, we selected the most two low JAG1 mRNA level cell lines, H1299 and H838, for exogenous expressing JAG1 and the most two high JAG1 mRNA level cell lines, HOP62 and H322M, for JAG1 silencing (S2 Fig). In H1299 and H838 cell lines, mRNA expression of HSPA2 was induced by JAG1 (S3 Fig). In HOP62 and H322M cell lines, mRNA expression of HSPA2 was reduced when JAG1 was silenced (S4 Fig). We further examined whether this JAG1/HSPA2 axis was independent from NOTCH signaling. Canonical proteolytic status of intracellular domain of NOTCH (NICD) family including NICD1, NICD2, NICD3, and NICD4 were analyzed in JAG1 manipulated cell lines (Fig 5). In addition, the transcriptional level of DLL1 and other NOTCH downstream molecules including CBF1, slug, snail1, SMAD3, HES1, HES3, HES5, HEY1, HEY2, and MYOD1 were also addressed. The results indicated that all NICDs and mRNA level of downstream molecules (except HES1) had no significant change in JAG1 overexpressed CL1-0 cells (Fig 5A). The similar results can be observed in JAG1 overexpressed H1299 (Fig 5B and S5 Fig) and H838 (Fig 5B and S6 Fig) cell lines or JAG1 silenced HOP62 and H322M cell lines (Fig 5C) when compared with control experiments. Only HES5 mRNA was affected by JAG1 in H1299 cell line. DAPT, a gamma-secretase inhibitor for blocking NOTCH signaling, was also unable to eliminate HSPA2 transcriptional up-regulation by JAG1 (S7 Fig). This supported the probable NOTCH-independent regulation at least in these cell lines. Finally, we would like to test whether JAG1 was colocalized with HSPA2 and fluorescent immunohistochemistry staining was performed in lung cancer patients’ tissue section. The result indicated JAG1 and HSPA2 were heterogeneously expressed in tumor parts and partially colocalized (S8 Fig). These results suggested that HSPA2 was a downstream gene of JAG1 and it also regulated tumor cell migration and invasion.

## Discussion

Discovery of oncogenes/tumor suppressor genes and characterization of the underlying molecular mechanisms not only clarify the complicated nature of tumorigenesis and cancer progression but also provide a basis for developing better strategies in cancer diagnosis, prognosis, prediction and therapy in the future. Our findings demonstrated the oncogenic role of JAG1 in lung cancer, promoting cancer invasion and metastasis. Clinical data showed that JAG1 mRNA expression is associated with poor survival in patients with subtype squamous carcinoma of NSCLC. In a previous study, the expression of JAG1 in lung tissues of idiopathic pulmonary fibrosis, which predisposes to lung cancer, was up-regulated. Moreover, the protein expression of JAG1 of squamous metaplastic lesions is positive in 83% cases assayed by immunohistochemical staining [19]. Therefore, JAG1 expression in lung cancers, including squamous carcinoma, may have high impact of clinical implications.

The Notch signaling pathways are usually considered to be ligand-induced [10], and the receptors and ligands are highly co-expressed in cancers [15, 20]. According to our microarray data, overexpression of JAG1 did not significantly turn on Notch signaling pathway (except NOTCH3 with 1.67-fold change) in CL1-0 cells (S4 Table). The result of real-time quantitative RT-PCR validation also provided similar conclusions (S5 Table). The equivocal expression of NOTCHs and downstream molecules in JAG1 transfected CL1-0 may result from the heterogeneous cell population. In addition, HES1 was slightly up-regulated in JAG1 overexpressed CL1-0 cells (Fig 5A and S5 Table). Thus, we cannot exclude the involvement of NOTCH signaling especially the NOTCH3 signaling in CL1-0. Notch ligands have been reported to have intrinsic ligand signaling activity independent of Notch receptors. After post-translational modifications, proteolytic processing, endocytosis, and membrane trafficking, Notch ligands would be multifunctional [21]. These implied that JAG1 in parts of NSCLC may induce noncanonical signaling pathways. In this study, we fully analyzed the NOTCH signaling in JAG1 overexpressed lung cancer cell lines by examining the proteolytic status of NICD and downstream molecules of NOTCH signaling. Only HES5 had found to be reduced in JAG1 overexpressed H1299 cell line (S5 Fig). Whether this had significance in tumor metastasis is still needed to be investigated. Taken together, our results indicated that JAG1 is also able to mediate cell migration, invasion and metastasis of NSCLC through a Notch-independent pathway.

Several studies have reported that JAG1 mediates multiple signaling pathways such as: AP-1, MAPK, EGFR, NF-κβ, and IL-6 in different tumor cells [22–26]. Our microarray data indicated that JAG1 modulates several genes such as Adhesion Molecule with Ig-Like Domain 2 (AMIGO2), Inhibin, Beta E (INHBE), Heat Shock 70kDa Protein 2 (HSPA2), Sperm Protein Associated with The Nucleus, X-Linked, Family Member (SPANX), and G Protein-Coupled Receptor, Class C, Group 5, Member B (GPRC5B). HSPA2 has been found to overexpress in many cancer types. Furthermore, patients with higher HSPA2 expression had shorter overall survival compared with lower HSPA2 patients [27–30]. These are consistent with our *in vitro* data that HSPA2 plays an oncogenic role in NSCLC cell lines. Importantly, we provided a novel depiction of the mechanism for HSPA2 signaling.

Although the underlying mechanism of HSPA2 in cancer metastasis was less documented, its critical role in tumor growth and metastatic potential had been assumed. Based on its involvement of formation of active CDC2/cycline B1 complex and expression in tumors, it may regulate cancer cell properties of malignancy such as migration and invasion [31–34]. Recently, HSPA2 was reported that it was expressed in SCC rather than in adenocarcinoma and correlated with poor overall survival, supporting our finding in SCC [29]. It was possible that HSPA2 correlated to poor survival in SCC due to the anti-apoptosis effect via its downstream factors such as lens epithelium-derived growth factor (LEDGF) [35]. Recently, heat shock protein 70 was also reported to interact with the NICD1 and contribute to NOTCH signaling [36]. In this study, we highlighted the role of JAG1 induced non-canonical Notch signaling in NSCLC. Although JAG1 and HSPA2 were only partially colocalized in patients’ tumor biopsy (S8 Fig), the regulation of HSPA2 by JAG1 could be an important find for lung cancer cell malignancy. Whether JAG1 can interact with receptors other than NOTCHs and induce HSPA2 transcriptional up-regulation needs to be further investigated. The possibility of HSPA2, a heat shock protein, to be a potential therapeutic target may be anticipated. In summary, our findings are the first to form the basis that JAG1 serves as a prognostic biomarker and JAG1/HSPA2 axis contributes to the lung cancer malignancy.

## Supporting Information

S1 Fig JAG1 expression and NSCLC patients’ survival. (PDF)

S2 Fig Endogenous JAG1 mRNA expression in six lung cancer cell lines. (PDF)

S3 Fig HSPA2 mRNA expression is up-regulated by JAG1 mRNA overexpression. (PDF)

S4 Fig HSPA2 mRNA expression is down-regulated by JAG1 mRNA silencing. (PDF)

S5 Fig mRNA expression of NOTCH downstream molecules in JAG1 overexpressed H1299 cells. (PDF)

S6 Fig mRNA expression of NOTCH downstream molecules in JAG1 overexpressed H838 cells. (PDF)

S7 Fig Up-regulation of HSPA2 by JAG1 was independent of NOTCH signaling. (PDF)

S8 Fig Fluorescent immunohistochemical staining for JAG1 and HSPA2. (PDF)

S1 Table Primer sequences of target genes used in SYBR Green real-time quantitative RT-PCR. (PDF)

S2 Table Top 10 differentially expressed gene in JAG1-overexpressed CL1-0 cells. (PDF)

S3 Table Validation of top 10 differentially expressed gene from Affymetrix microarray analysis in JAG1-overexpressed or JAG1-silenced CL1-0, CL1-5, A549 and H226 cells by real-time quantitative RT-PCR. (PDF)

S4 Table Notch-related genes expression assayed by Affymetrix microarray analysis (PDF)

S5 Table Notch-related genes expression assayed by real-time quantitative RT-PCR. (PDF)

S1 Text Supplementary Methods. (DOCX)

## References

[1] Siegel RL, Miller KD, Jemal A. Cancer statistics, 2015. CA: a cancer journal for clinicians. 2015;65(1):5–29. 10.3322/caac.21254 . 25559415

[2] Hanahan D, Weinberg RA. Hallmarks of cancer: the next generation. Cell. 2011;144(5):646–74. 10.1016/j.cell.2011.02.013 . 21376230

[3] Chin L, Andersen JN, Futreal PA. Cancer genomics: from discovery science to personalized medicine. Nature medicine. 2011;17(3):297–303. 10.1038/nm.2323 . 21383744

[4] Jones PA, Baylin SB. The epigenomics of cancer. Cell. 2007;128(4):683–92. 10.1016/j.cell.2007.01.029 17320506 PMC3894624

[5] Chu YW, Yang PC, Yang SC, Shyu YC, Hendrix MJ, Wu R, et al Selection of invasive and metastatic subpopulations from a human lung adenocarcinoma cell line. American journal of respiratory cell and molecular biology. 1997;17(3):353–60. 10.1165/ajrcmb.17.3.2837 . 9308922

[6] Chang TP, Yu SL, Lin SY, Hsiao YJ, Chang GC, Yang PC, et al Tumor suppressor HLJ1 binds and functionally alters nucleophosmin via activating enhancer binding protein 2alpha complex formation. Cancer research. 2010;70(4):1656–67. 10.1158/0008-5472.CAN-09-2453 . 20145123

[7] Chen CC, Chen HY, Su KY, Hong QS, Yan BS, Chen CH, et al Shisa3 is associated with prolonged survival through promoting beta-catenin degradation in lung cancer. American journal of respiratory and critical care medicine. 2014;190(4):433–44. 10.1164/rccm.201312-2256OC . 25036006

[8] Chen CH, Chuang SM, Yang MF, Liao JW, Yu SL, Chen JJ. A novel function of YWHAZ/beta-catenin axis in promoting epithelial-mesenchymal transition and lung cancer metastasis. Molecular cancer research: MCR. 2012;10(10):1319–31. 10.1158/1541-7786.MCR-12-0189 . 22912335

[9] Chen HW, Lee JY, Huang JY, Wang CC, Chen WJ, Su SF, et al Curcumin inhibits lung cancer cell invasion and metastasis through the tumor suppressor HLJ1. Cancer research. 2008;68(18):7428–38. 10.1158/0008-5472.CAN-07-6734 . 18794131

[10] Hori K, Sen A, Artavanis-Tsakonas S. Notch signaling at a glance. Journal of cell science. 2013;126(Pt 10):2135–40. 10.1242/jcs.127308 23729744 PMC3672934

[11] Li L, Krantz ID, Deng Y, Genin A, Banta AB, Collins CC, et al Alagille syndrome is caused by mutations in human Jagged1, which encodes a ligand for Notch1. Nature genetics. 1997;16(3):243–51. 10.1038/ng0797-243 . 9207788

[12] Oda T, Elkahloun AG, Pike BL, Okajima K, Krantz ID, Genin A, et al Mutations in the human Jagged1 gene are responsible for Alagille syndrome. Nature genetics. 1997;16(3):235–42. 10.1038/ng0797-235 . 9207787

[13] Fiaschetti G, Schroeder C, Castelletti D, Arcaro A, Westermann F, Baumgartner M, et al NOTCH ligands JAG1 and JAG2 as critical pro-survival factors in childhood medulloblastoma. Acta neuropathologica communications. 2014;2:39 10.1186/2051-5960-2-39 24708907 PMC4023630

[14] Dai Y, Wilson G, Huang B, Peng M, Teng G, Zhang D, et al Silencing of Jagged1 inhibits cell growth and invasion in colorectal cancer. Cell death & disease. 2014;5:e1170 10.1038/cddis.2014.137 . 24722295 PMC5424114

[15] Reedijk M, Odorcic S, Chang L, Zhang H, Miller N, McCready DR, et al High-level coexpression of JAG1 and NOTCH1 is observed in human breast cancer and is associated with poor overall survival. Cancer research. 2005;65(18):8530–7. 10.1158/0008-5472.CAN-05-1069 . 16166334

[16] Tran QN. A novel method for finding non-small cell lung cancer diagnosis biomarkers. BMC medical genomics. 2013;6 Suppl 1:S11 10.1186/1755-8794-6-S1-S11 23369236 PMC3552706

[17] Tsai MF, Wang CC, Chang GC, Chen CY, Chen HY, Cheng CL, et al A new tumor suppressor DnaJ-like heat shock protein, HLJ1, and survival of patients with non-small-cell lung carcinoma. Journal of the National Cancer Institute. 2006;98(12):825–38. 10.1093/jnci/djj229 . 16788156

[18] Chen JJ, Peck K, Hong TM, Yang SC, Sher YP, Shih JY, et al Global analysis of gene expression in invasion by a lung cancer model. Cancer research. 2001;61(13):5223–30. . 11431363

[19] Murata K, Ota S, Niki T, Goto A, Li CP, Ruriko UM, et al p63—Key molecule in the early phase of epithelial abnormality in idiopathic pulmonary fibrosis. Experimental and molecular pathology. 2007;83(3):367–76. 10.1016/j.yexmp.2007.03.006 . 17498688

[20] Reedijk M, Odorcic S, Zhang H, Chetty R, Tennert C, Dickson BC, et al Activation of Notch signaling in human colon adenocarcinoma. International journal of oncology. 2008;33(6):1223–9. 19020755 10.3892/ijo_00000112 PMC2739737

[21] D'Souza B, Miyamoto A, Weinmaster G. The many facets of Notch ligands. Oncogene. 2008;27(38):5148–67. 10.1038/onc.2008.229 18758484 PMC2791526

[22] LaVoie MJ, Selkoe DJ. The Notch ligands, Jagged and Delta, are sequentially processed by alpha-secretase and presenilin/gamma-secretase and release signaling fragments. The Journal of biological chemistry. 2003;278(36):34427–37. 10.1074/jbc.M302659200 . 12826675

[23] Zeng Q, Li S, Chepeha DB, Giordano TJ, Li J, Zhang H, et al Crosstalk between tumor and endothelial cells promotes tumor angiogenesis by MAPK activation of Notch signaling. Cancer cell. 2005;8(1):13–23. 10.1016/j.ccr.2005.06.004 . 16023595

[24] Sansone P, Storci G, Tavolari S, Guarnieri T, Giovannini C, Taffurelli M, et al IL-6 triggers malignant features in mammospheres from human ductal breast carcinoma and normal mammary gland. The Journal of clinical investigation. 2007;117(12):3988–4002. 18060036 10.1172/JCI32533 PMC2096439

[25] Choi K, Ahn YH, Gibbons DL, Tran HT, Creighton CJ, Girard L, et al Distinct biological roles for the notch ligands Jagged-1 and Jagged-2. The Journal of biological chemistry. 2009;284(26):17766–74. 10.1074/jbc.M109.003111 19398556 PMC2719415

[26] Wang Z, Li Y, Banerjee S, Kong D, Ahmad A, Nogueira V, et al Down-regulation of Notch-1 and Jagged-1 inhibits prostate cancer cell growth, migration and invasion, and induces apoptosis via inactivation of Akt, mTOR, and NF-kappaB signaling pathways. Journal of cellular biochemistry. 2010;109(4):726–36. 10.1002/jcb.22451 . 20052673

[27] Zhang H, Gao H, Liu C, Kong Y, Wang C, Zhang H. Expression and clinical significance of HSPA2 in pancreatic ductal adenocarcinoma. Diagnostic pathology. 2015;10:13 10.1186/s13000-015-0253-9 25890028 PMC4383074

[28] Fu Y, Zhao H, Li XS, Kang HR, Ma JX, Yao FF, et al Expression of HSPA2 in human hepatocellular carcinoma and its clinical significance. Tumour biology: the journal of the International Society for Oncodevelopmental Biology and Medicine. 2014;35(11):11283–7. 10.1007/s13277-014-2430-y . 25117073

[29] Scieglinska D, Gogler-Piglowska A, Butkiewicz D, Chekan M, Malusecka E, Harasim J, et al HSPA2 is expressed in human tumors and correlates with clinical features in non-small cell lung carcinoma patients. Anticancer research. 2014;34(6):2833–40. . 24922646

[30] Zhang H, Chen W, Duan CJ, Zhang CF. Overexpression of HSPA2 is correlated with poor prognosis in esophageal squamous cell carcinoma. World journal of surgical oncology. 2013;11:141 10.1186/1477-7819-11-141 23777267 PMC3698197

[31] Dix DJ, Allen JW, Collins BW, Mori C, Nakamura N, Poorman-Allen P, et al Targeted gene disruption of Hsp70-2 results in failed meiosis, germ cell apoptosis, and male infertility. Proc Natl Acad Sci U S A. 1996;93(8):3264–8. 8622925 10.1073/pnas.93.8.3264 PMC39594

[32] Garg M, Kanojia D, Seth A, Kumar R, Gupta A, Surolia A, et al Heat-shock protein 70–2 (HSP70-2) expression in bladder urothelial carcinoma is associated with tumour progression and promotes migration and invasion. Eur J Cancer. 2010;46(1):207–15. 10.1016/j.ejca.2009.10.020 19914824

[33] Rohde M, Daugaard M, Jensen MH, Helin K, Nylandsted J, Jaattela M. Members of the heat-shock protein 70 family promote cancer cell growth by distinct mechanisms. Genes Dev. 2005;19(5):570–82. 10.1101/gad.305405 15741319 PMC551577

[34] Zhu D, Dix DJ, Eddy EM. HSP70-2 is required for CDC2 kinase activity in meiosis I of mouse spermatocytes. Development. 1997;124(15):3007–14. . 9247342 10.1242/dev.124.15.3007

[35] Daugaard M, Kirkegaard-Sorensen T, Ostenfeld MS, Aaboe M, Hoyer-Hansen M, Orntoft TF, et al Lens epithelium-derived growth factor is an Hsp70-2 regulated guardian of lysosomal stability in human cancer. Cancer Res. 2007;67(6):2559–67. 10.1158/0008-5472.CAN-06-4121 . 17363574

[36] Jurynczyk M, Lewkowicz P, Domowicz M, Mycko MP, Selmaj KW. Heat shock protein 70 (Hsp70) interacts with the Notch1 intracellular domain and contributes to the activity of Notch signaling in myelin-reactive CD4 T cells. J Neuroimmunol. 2015;287:19–26. 10.1016/j.jneuroim.2015.08.007 . 26439956

