# Supplementary material for: JAG1 Is Associated with Poor Survival through Inducing Metastasis in Lung Cancer
Source: PLoS One. 2016 Mar 1;11(3):e0150355. doi: 10.1371/journal.pone.0150355 (PMC4773101; doi:10.1371/journal.pone.0150355)

**S1 Fig. JAG1 expression and NSCLC patients' survival.**

JAG1 mRNA level and overall survival of NSCLC patients (n=90). All statistical tests were two sided, and $p$ <0.05 was considered to be statistically significant.

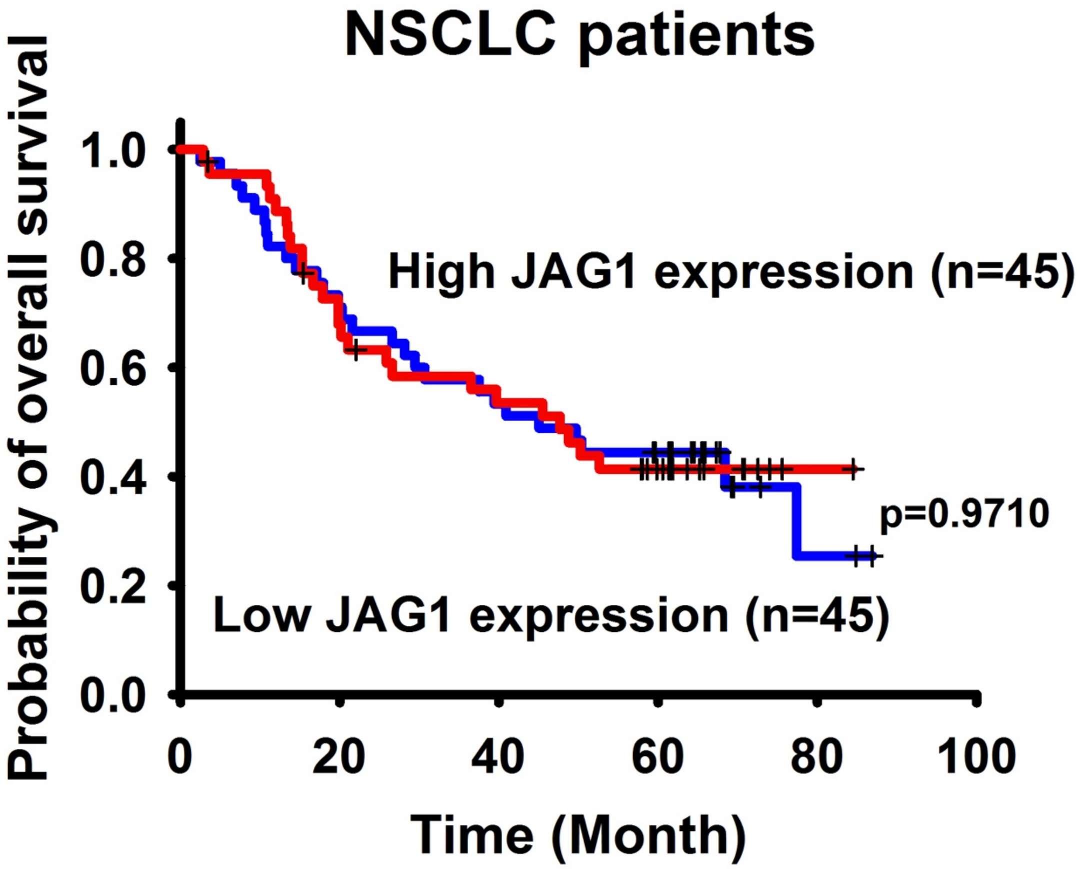

Supplement: S1 Fig — (PDF) [file pone.0150355.s001.pdf]
