# Supplementary material for: JAG1 Is Associated with Poor Survival through Inducing Metastasis in Lung Cancer
Source: PLoS One. 2016 Mar 1;11(3):e0150355. doi: 10.1371/journal.pone.0150355 (PMC4773101; doi:10.1371/journal.pone.0150355)

S2 Fig. Endogenous JAG1 mRNA expression in six lung cancer cell lines.

JAG1 was transiently overexpressed in six cell lines. JAG1 mRNA was measured by real-time quantitative RT-PCR and normalized to TBP.

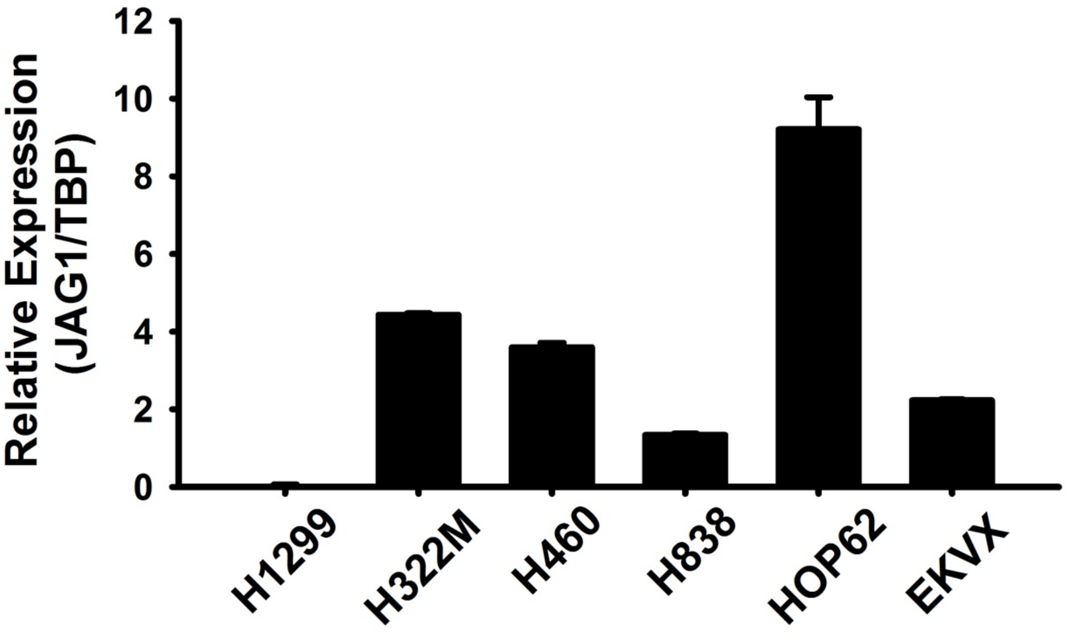

Supplement: S2 Fig — (PDF) [file pone.0150355.s002.pdf]
