# Supplementary material for: JAG1 Is Associated with Poor Survival through Inducing Metastasis in Lung Cancer
Source: PLoS One. 2016 Mar 1;11(3):e0150355. doi: 10.1371/journal.pone.0150355 (PMC4773101; doi:10.1371/journal.pone.0150355)

S3 Fig. HSPA2 mRNA expression is up-regulated by JAG1 mRNA overexpression.

Left, JAG1 was transiently overexpressed in H1299 and H838 cell lines. Right, HSPA2 mRNA was measured by real-time quantitative RT-PCR and normalized to TBP.

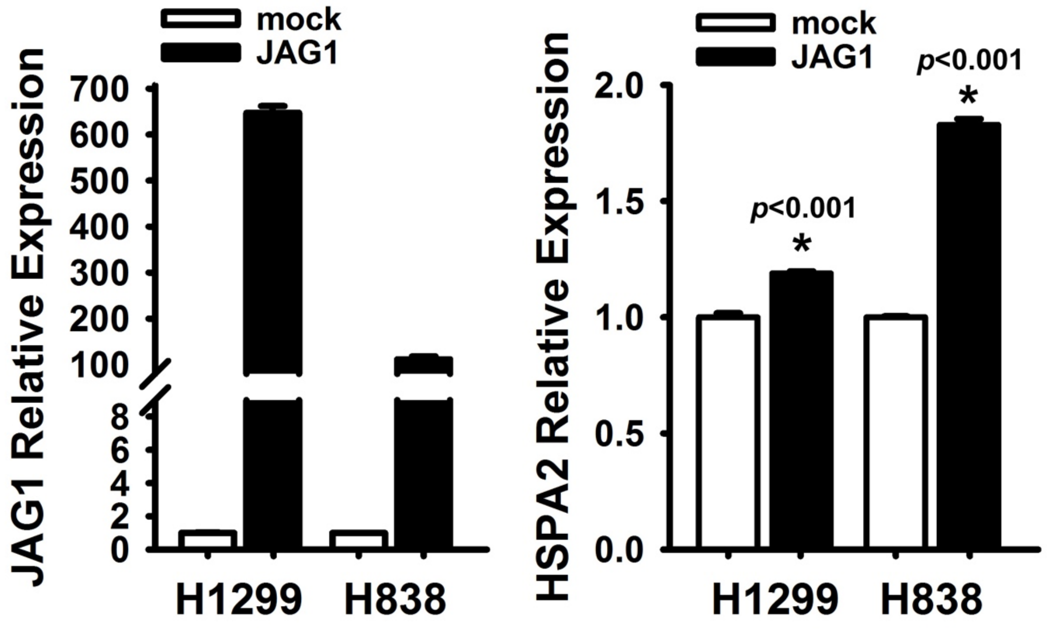

Supplement: S3 Fig — (PDF) [file pone.0150355.s003.pdf]
