# Supplementary material for: JAG1 Is Associated with Poor Survival through Inducing Metastasis in Lung Cancer
Source: PLoS One. 2016 Mar 1;11(3):e0150355. doi: 10.1371/journal.pone.0150355 (PMC4773101; doi:10.1371/journal.pone.0150355)

S4 Fig. HSPA2 mRNA expression is down-regulated by JAG1 mRNA silencing.

Left, JAG1 mRNA level was silenced by two independent specific siRNA in HOP62 and H322M cell lines. Right, HSPA2 mRNA was measured by real-time quantitative RT-PCR and normalized to TBP.

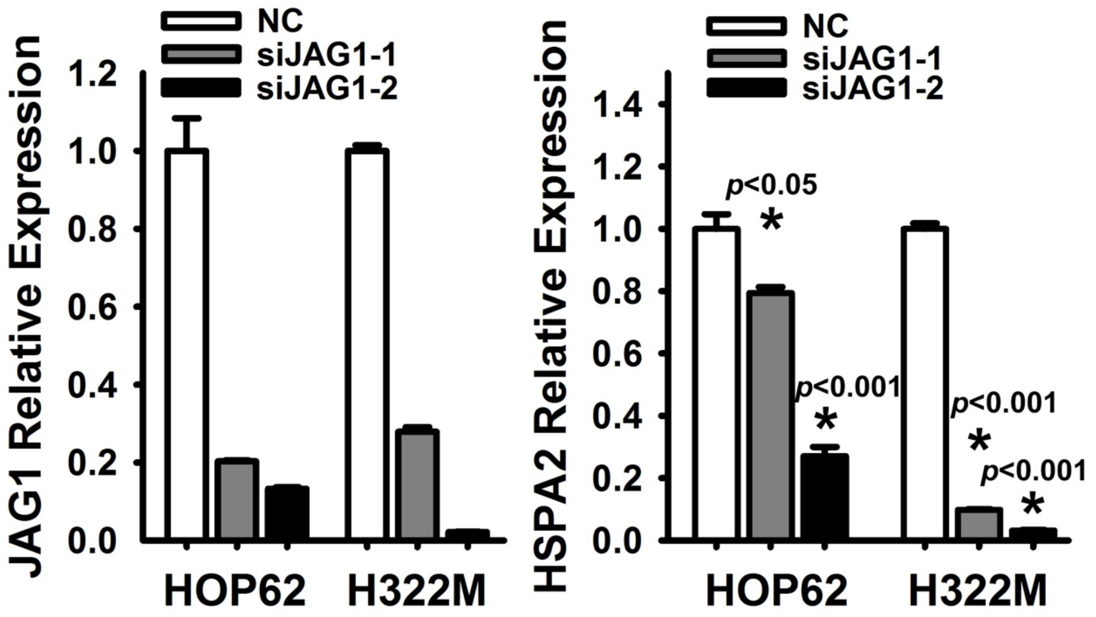

Supplement: S4 Fig — (PDF) [file pone.0150355.s004.pdf]
