# Supplementary material for: JAG1 Is Associated with Poor Survival through Inducing Metastasis in Lung Cancer
Source: PLoS One. 2016 Mar 1;11(3):e0150355. doi: 10.1371/journal.pone.0150355 (PMC4773101; doi:10.1371/journal.pone.0150355)

S6 Fig. mRNA expression of NOTCH downstream molecules in JAG1 overexpressed H838 cells.

Analysis of NOTCH downstream molecules including CBF1, slug, snail1, SMAD3, HES1, HES3, HES5, HEY1, HEY2, and MYOD1 mRNA expression as well as DLL1 in JAG1 overexpressing H838 cell line by real-time quantitative RT-PCR and normalized to mock transfectants.

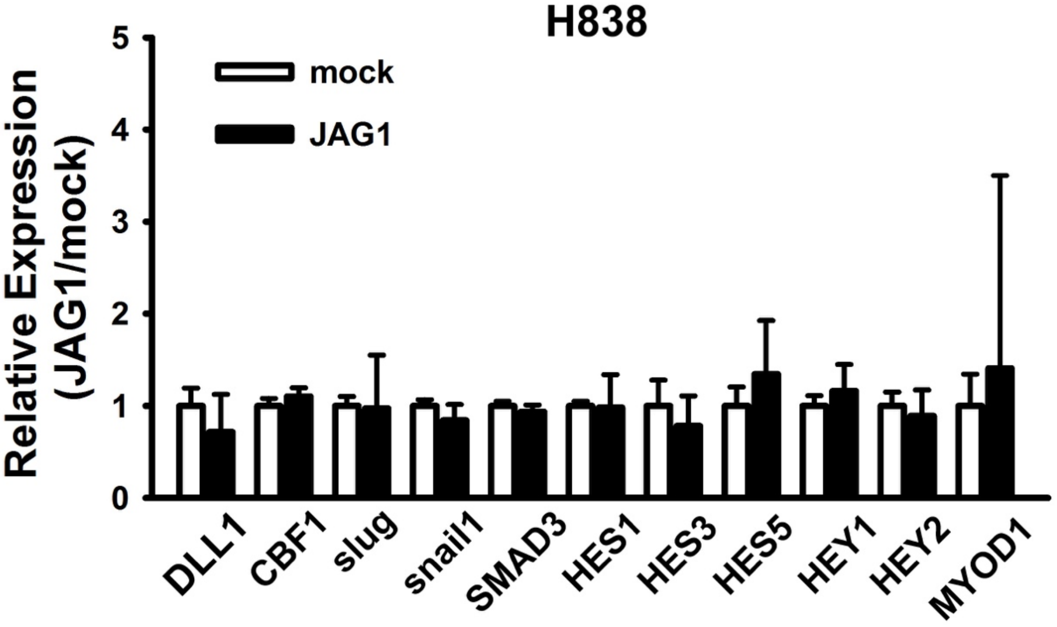

Supplement: S6 Fig — (PDF) [file pone.0150355.s006.pdf]
