# Supplementary material for: JAG1 Is Associated with Poor Survival through Inducing Metastasis in Lung Cancer
Source: PLoS One. 2016 Mar 1;11(3):e0150355. doi: 10.1371/journal.pone.0150355 (PMC4773101; doi:10.1371/journal.pone.0150355)

S7 Fig. Up-regulation of HSPA2 by JAG1 was independent of NOTCH signaling.

Analysis of mRNA transcriptional level of HSPA2 in JAG1 transfected cell lines (CL1-0, H1299, and H838) with or without DAPT, a gamma-secretase inhibitor for blocking NOTCH signaling.

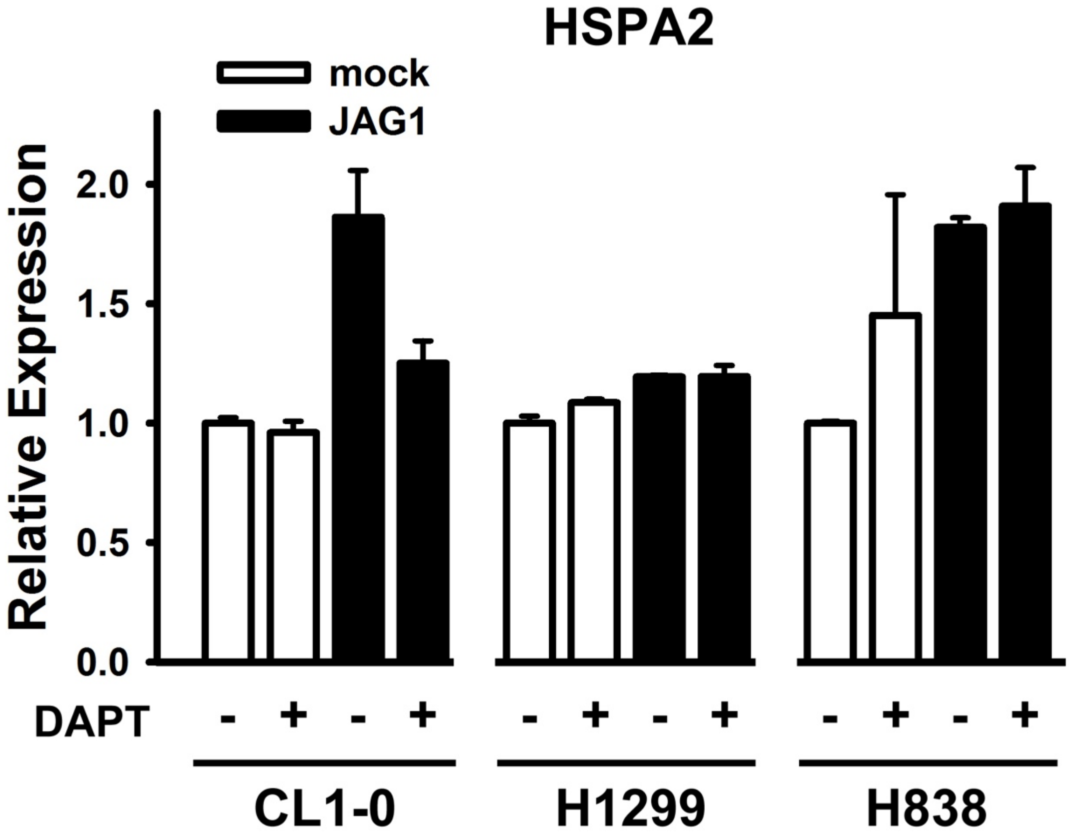

Supplement: S7 Fig — (PDF) [file pone.0150355.s007.pdf]
