# Supplementary material for: JAG1 Is Associated with Poor Survival through Inducing Metastasis in Lung Cancer
Source: PLoS One. 2016 Mar 1;11(3):e0150355. doi: 10.1371/journal.pone.0150355 (PMC4773101; doi:10.1371/journal.pone.0150355)

S8 Fig. Fluorescent immunohistochemical staining for JAG1 and HSPA2.

FFPE tumor sections from patients with lung adenocarcinoma were performed standard fluorescent immunohistochemical staining followed by confocal microscope analysis. Green fluorescence indicated the expression pattern for JAG1 while red fluorescence represented the expression pattern for HSPA2. T, tumor parts.

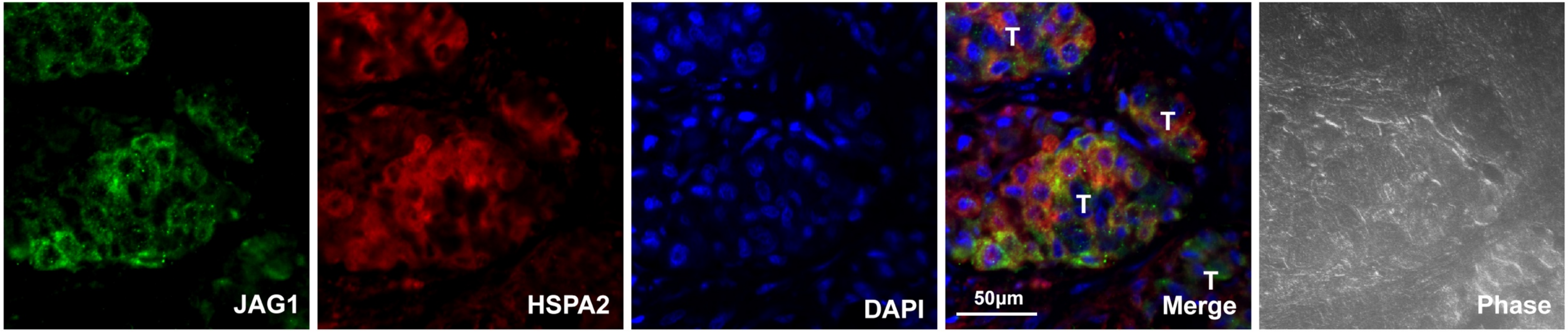

Supplement: S8 Fig — (PDF) [file pone.0150355.s008.pdf]
