# Supplementary material for: JAG1 Is Associated with Poor Survival through Inducing Metastasis in Lung Cancer
Source: PLoS One. 2016 Mar 1;11(3):e0150355. doi: 10.1371/journal.pone.0150355 (PMC4773101; doi:10.1371/journal.pone.0150355)
Supplement: S1 Table — (PDF) [file pone.0150355.s009.pdf]

S1 Table. Primer sequences of target genes used in SYBER Green real-time quantitative PCR

Gene	Forward 5'-->3'	Reverse 5'-->3'
AMIGO2	GGCTCTCCCAGTTGCAGAAA	AACATCAGTTCTGCCAGCTTGA
AP1	GCTGGAGGAAAAAGTGAAAACCT	GTTGGCACCCACTGTTAACGT
ASCL1	CCCAACTACTCCAACGACTTGAA	AACCAGTTGGTGAAGTCGAGAAG
CEBPA	CCCAACTACTCCAACGACTTGAA	AACCAGTTGGTGAAGTCGAGAAG
DLL	TGGCAAGACTCCCGTTTCTC	CTCGGTCTGAACTCGGTTTCTC
F2RL1	CATTCCTGCCCTGAACATCA	AGGAAGGCTGGGAACAGAAAG
GPER	GGAGCACCAGCAGTACGTGAT	TTCTCGCGGAAGCTGATGTT
GPRC5B	CAATGCAGCTCTCCGAACAG	ACGACGGCCATCTCAGTTG
HES1	GAAGGCGGACATTCTGGAAA	AGCGCAGCCGTCATCTG
HES3	TCGCTGGAGAAACACTA	GGAGATGAGGTCGGCAGC
HES5	GCAGCATCGAGCAGCTGAA	ACGAAGGCTTTGCTGTGCTT
HEY1	AGAAGGCTGGTACCCAGTGCTTT	AGTAACCTTTCCCTCCTGCCGT
HEY2	AGACTTGTGCCAACTGCTTTTG	CAAAGTAGCCTTTACCCCCTGTT
HSPA2	GCACCGGTAAGGAAAACAAA	AAAACGCCCTGGAGTCCTAT
INHBE	ACACCAGCAGCCCTTCCTAGA	TAATGGTCTCGCCTGCAACA
JAG1	ACCGCAACCGCATCGT	AATCCCACGCCTCCACAA
MYOD1	CCCTCCCAACAGCGCTTTA	AAGGGTGCTGCGTGGAAA
NOTCH1	GTCAACGCCGTAGATGACC	TTGTTAGCCCCGTTCTTCAG
NOTCH2	AGTGGCAGTGCTGGAAGCTT	AGGACCATACCAAACATCTCATTGT
NOTCH3	TGATCGGCTCGGTAGTAATGC	GACAACGCTCCCAGGTAGTCA
NOTCH4	CAGGGACATGGTGTACCCCTAT	CACCACAAACCCAGCACTGA
Opsin	GCTGGCCTATGAACGTTACATTC	CGCCAGTGAGTAGAGCCAGAT
RBP-J kappa (CBF1)	TTCACAGTCCGAGATGGCTACAT	CAATAATGCGGTCTGCTTATCAAC
SLUG	TAGCATATTCGGACCCACACATT	AGATTTGACCTGTCTGCAA?TGC
SMAD3	GACGAGGTCTGCGTGAATCC	TGCGTCCATGCTGTGGTT
SNAIL1	CTTCCCATGGCCATTTCTGT	GCTCAAAGCAGCTGTATCCAAAC
SPANXA	AACGAGGCCAACGAGATGAT	CTAGTATGGTCGAGGACTCAGATGTT
TBP (internal control)	CACGAACCACGGCACTGATT	TTTTCTTGCTGCCAGTCTGGAC
