# Supplementary material for: JAG1 Is Associated with Poor Survival through Inducing Metastasis in Lung Cancer
Source: PLoS One. 2016 Mar 1;11(3):e0150355. doi: 10.1371/journal.pone.0150355 (PMC4773101; doi:10.1371/journal.pone.0150355)
Supplement: S2 Table — (PDF) [file pone.0150355.s010.pdf]

S2 Table. Top 10 differentially expressed gene in JAG1-expressed CL1-0 cells

**RefSeq ID**	**Gene Symbol**	**Fold Change (JAG1/Mock*)**
NM_181847	AMIGO2	9.21
NM_021979	HSPA2	4.18
NM_013453	SPANX	3.27
NM_016235	GPRC5B	2.73
NM_005242	F2RL1	2.48
NM_001039966	GPER	2.37
NM_014322	Opsin	2.14
NM_004364	CEBP	0.47
NM_002228	AP1	0.44
NM_031479	INHBE	0.19

* Gene fold change in JAG1 transfectants compared with mock control assayed by Affymetrix microarray analysis
