# Supplementary material for: JAG1 Is Associated with Poor Survival through Inducing Metastasis in Lung Cancer
Source: PLoS One. 2016 Mar 1;11(3):e0150355. doi: 10.1371/journal.pone.0150355 (PMC4773101; doi:10.1371/journal.pone.0150355)
Supplement: S3 Table — (PDF) [file pone.0150355.s011.pdf]

S3 Table. Validation of top 10 differentially expressed gene from Affymetrix microarray analysis in JAG1-overexpressed or JAG1-silenced CL1-0, CL1-5, A549 and H226 cells by real-time quantitative RT-PCR

RefSeq ID	Gene Symbol	Fold Change				
		JAG1/Mock			siJAG1-1/NC	siJAG1-2/NC
		CL1-0	A549	H226	CL1-5	
NM_181847	AMIGO2	1.09	1.17	1.11	0.76	0.98
NM_021979	HSPA2	1.40	1.35	1.58	0.76	0.80
NM_013453	SPANX	0.72	1.97	1.14	0.53	1.25
NM_016235	GPRC5B	2.61	1.02	0.99	0.44	0.76
NM_005242	F2RL1	1.14	1.20	1.12	0.89	1.11
NM_001039966	GPER	0.99	2.96	1.30	1.53	1.15
NM_014322	Opsin	0.92	1.33	0.86	0.64	NA
NM_004364	CEBP	0.66	1.44	1.00	0.94	NA
NM_002228	AP1	0.81	1.29	1.12	1.13	NA
NM_031479	INHBE	0.86	3.15	1.39	1.06	0.88
