# Supplementary material for: JAG1 Is Associated with Poor Survival through Inducing Metastasis in Lung Cancer
Source: PLoS One. 2016 Mar 1;11(3):e0150355. doi: 10.1371/journal.pone.0150355 (PMC4773101; doi:10.1371/journal.pone.0150355)
Supplement: S4 Table — (PDF) [file pone.0150355.s012.pdf]

S4 Table. Notch-related genes expression assayed by Affymetrix microarray analysis

**RefSeq ID**	**Gene Symbol**	**Fold Change (JAG1/Mock*)**
NM_004316	ASCL1	1.00
NM_005524	HES1	0.88
NM_001040708	HEY1	0.83
NM_003068	SLUG	1.00
NM_017617	NOTCH1	1.00
NM_024408	NOTCH2	1.30
NM_000435	NOTCH3	1.67
NM_004557	NOTCH4	0.75

* Gene fold change in JAG1 transfectants compared with mock control assayed by Affymetrix microarray analysis
