# Supplementary material for: JAG1 Is Associated with Poor Survival through Inducing Metastasis in Lung Cancer
Source: PLoS One. 2016 Mar 1;11(3):e0150355. doi: 10.1371/journal.pone.0150355 (PMC4773101; doi:10.1371/journal.pone.0150355)
Supplement: S5 Table — (PDF) [file pone.0150355.s013.pdf]

S5 Table. Notch-related genes expression assayed by real-time quantitative RT-PCR

RefSeq ID	Gene Symbol	Fold Change				
		JAG1/Mock			siJAG1-1/NC	siJAG1-2/NC
		CL1-0	A549	H226	CL1-5	
NM_004316	ASCL1	0.93±0.11	0.64±0.17	0.82±0.08	0.87±0.06	0.80±0.24
NM_005524	HES1	1.27±0.12	1.07±0.07	0.80±0.09	0.84±0.04	1.24±0.04
NM_001040708	HEY1	0.88±0.04	1.02±0.18	1.27±0.18	0.59±0.03	0.72±0.10
NM_003068	SLUG	0.96±0.02	1.10±0.19	0.74±0.13	0.86±0.14	1.37±0.23
NM_017617	NOTCH1	0.97±0.07	1.01±0.07	0.87±0.05	0.99±0.03	1.05±0.19
NM_024408	NOTCH2	1.46±0.23	1.04±0.11	0.74±0.04	0.84±0.01	1.00±0.09
NM_000435	NOTCH3	2.23±0.18	1.00±0.14	1.23±0.12	0.86±0.09	0.63±0.31
NM_004557	NOTCH4	0.78±0.09	0.11±0.04	0.55±0.05	0.62±0.16	1.08±0.37
