# Supplementary material for: JAG1 Is Associated with Poor Survival through Inducing Metastasis in Lung Cancer
Source: PLoS One. 2016 Mar 1;11(3):e0150355. doi: 10.1371/journal.pone.0150355 (PMC4773101; doi:10.1371/journal.pone.0150355)
Supplement: S1 Text — (DOCX) [file pone.0150355.s014.docx]

**S1 Text. Supplementary Methods**

**Identification of JAG1 downstream genes by microarray analysis**

Expression of downstream genes of JAG1 was identified using Affymetrix oligonucleotide microarray analysis (HG-U133 plus 2). Totally 83 genes were differentially expressed with greater than two-fold change under false discovery rate protection (FDR< 0.05) in JAG1 transfectants (CL1-0/JAG1) as compared to mock transfectants (CL1-0/vector control) (S2 Table).

**High expression of JAG1 versus low expression of JAG1 of NSCLC patients**

Real time quantitative RT-PCR was used for quantifying transcriptional expression of JAG1. The threshold cycle (Ct) was defined as the fractional cycle number at which the fluorescence generated by cleavage of the probe exceeds a fixed threshold above baseline. In this study, we used TBP mRNA as an internal control. The expression of JAG1 was normalized against that of TBP and was expressed as -ΔCt = -[Ct_JAG1_-Ct_TBP_]. The ratio of JAG1 to TBP was calculated as 2-ΔCt *K, where K is a constant. The median value of –ΔCt in the 35 subtype squamous carcinoma is 2.34. The median value was used to classify patients as JAG1 high-expression or JAG1 low-expression group.

**Fluorescent immunohistochemistry**

Ten micron-thick FFPE sections from tumor biopsy of lung cancer patients were de-paraffin according to standard procedure followed by using boiled sodium citrate buffer for antigen retrieval. After blocking with 5% fetal bovine serum (FBS) and 0.5% Tween-20 in PBS (FBS/PBST), tissue sections were incubated overnight with individual primary antibodies against Jagged1 (1:200) (#sc-6011, Santa Cruz) and HSPA2 (1:200) (GTX110079, GeneTex) in FBS/PBST at 4^o^C. Unbound antibodies were removed by washing in PBST three times followed by detection with an Alexa Fluor 488 conjugated anti-goat or Cy3 conjugated anti-rabbit second antibodies at room temperature for 1 h. After staining with DAPI and washing off unbound materials, sections were air-dried, cover-slipped with mounting medium (DakoCytomation, Denmark) and examined on NIKON D-ECLIPSE C1si confocal microscope.
